# Insights into the Management of Large Carnivores for Profitable Wildlife-Based Land Uses in African Savannas

**DOI:** 10.1371/journal.pone.0059044

**Published:** 2013-03-20

**Authors:** Paul J. Funston, Rosemary J. Groom, Peter A. Lindsey

**Affiliations:** 1 Department of Nature Conservation, Tshwane University of Technology, Pretoria, South Africa; 2 Department of Zoology, University of Johannesburg, Auckland Park, South Africa; 3 African Wildlife Conservation Fund, Doral, Florida, United States of America; 4 Mammal Research Institute, University of Pretoria, Pretoria, South Africa; 5 Panthera Foundation, New York City, New York, United States of America; Australian Wildlife Conservancy, Australia

## Abstract

Large African predators, especially lions (*Panthera leo*) and leopards (*Panthera pardus*), are financially valuable for ecotourism and trophy hunting operations on land also utilized for the production of other wildlife species for the same purpose. Predation of ungulates used for trophy hunting can create conflict with landholders and trade off thus exists between the value of lions and leopards and their impact on ungulate populations. Therefore productionist and conservation trade-offs are complexly graded and difficult to resolve. We investigated this with a risk-benefit analysis on a large private wildlife production area in Zimbabwe. Our model showed that lions result in substantial financial costs through predation on wild ungulates that may not be offset by profits from hunting them, whereas the returns from trophy hunting of leopards are projected to exceed the costs due to leopard predation. In the absence of additional income derived from photo-tourism the number of lions may need to be managed to minimize their impact. Lions drive important ecological processes, but there is a need to balance ecological and financial imperatives on wildlife ranches, community wildlife lands and other categories of multiple use land used for wildlife production. This will ensure the competitiveness of wildlife based land uses relative to alternatives. Our findings may thus be limited to conservancies, community land-use areas and commercial game ranches, which are expansive in Africa, and should not necessarily applied to areas where biodiversity conservation is the primary objective, even if hunting is allowed there.

## Introduction

In Africa, wildlife occurs in a variety of scenarios, including *inter alia* fully protected lands and state, private and communally owned lands were wild animals are utilized commercially as a form of land use in accompaniment to, or as a replacement for traditional livestock farming. Where wildlife is utilized commercially, there can be conflicts among different forms of wildlife-use and between financial and conservation objectives [1], which can create tension that is not necessarily easy to resolve. For example, financial objectives may require generation of maximal incomes from hunting and photo-tourism, whereas conservation objectives may require restoration and maintenance of natural biological processes and ecological interactions. Land managers often attempt to achieve financial and conservation objectives in the same area. This often requires balancing various trade-offs, some of which may be complexly graded. For example, large carnivores such as lions (*Panthera leo*) and leopards (*Panthera pardus*) have potential to generate significant returns from both photo-tourism and trophy hunting [2], [3]. However, those same species can impose significant costs by consuming herbivores that could otherwise be hunted as trophies or for meat [4], [5], [6]. In some cases, such conflict results in efforts to control carnivore numbers, or even eradicate species perceived to be particularly problematic. Thus while hunting and wildlife-ranching provide important economic incentives for conserving wild ungulates on private land, the benefits of such land uses for large predators are more varied [7]. Depending on the extent to which ecotourism is practised, the proportion of the sustainable yield of wild ungulates that is utilized by ranchers, the prey profile of carnivores (and value of species affected by predation), and whether land owners are able to access permits for hunting lions and leopards, large carnivores can either confer net financial benefits or net costs.

At natural densities large African carnivores, especially lions, generally limit ungulate populations [8], [9], [10]. Consequently, the number of herbivores that can be sustainably removed by trophy hunting or live capture is limited by the relative numbers of large carnivores in a given area [4], and the number of carnivores that can be hunted is largely influenced by the social and breeding behaviour of the respective carnivores [3]. Additionally, wildlife populations are impacted by poaching for meat in many areas, further reducing sustainable legal harvests [11].

In many African countries, large areas of land are designated hunting concessions or multiple-use zones, where trophy hunting is practised [12], [13]. Such areas provide scope for conserving lions, and other large predators, even when they are hunted [14]. The success with which such species are conserved in those areas depends on the land tenure system and governance in place, the extent to which earnings from hunting translate into incentives for wildlife conservation, and also on land-use objectives. In this paper we assess the trade-offs between conservation and financial objectives as related to the management of large carnivores in a study site in south east Zimbabwe that supports the full suite of indigenous large carnivores, including a globally significant population of endangered African wild dogs (*Lycaon pictus*) [11].

## Results

### Population Estimates

**Figure 1:**
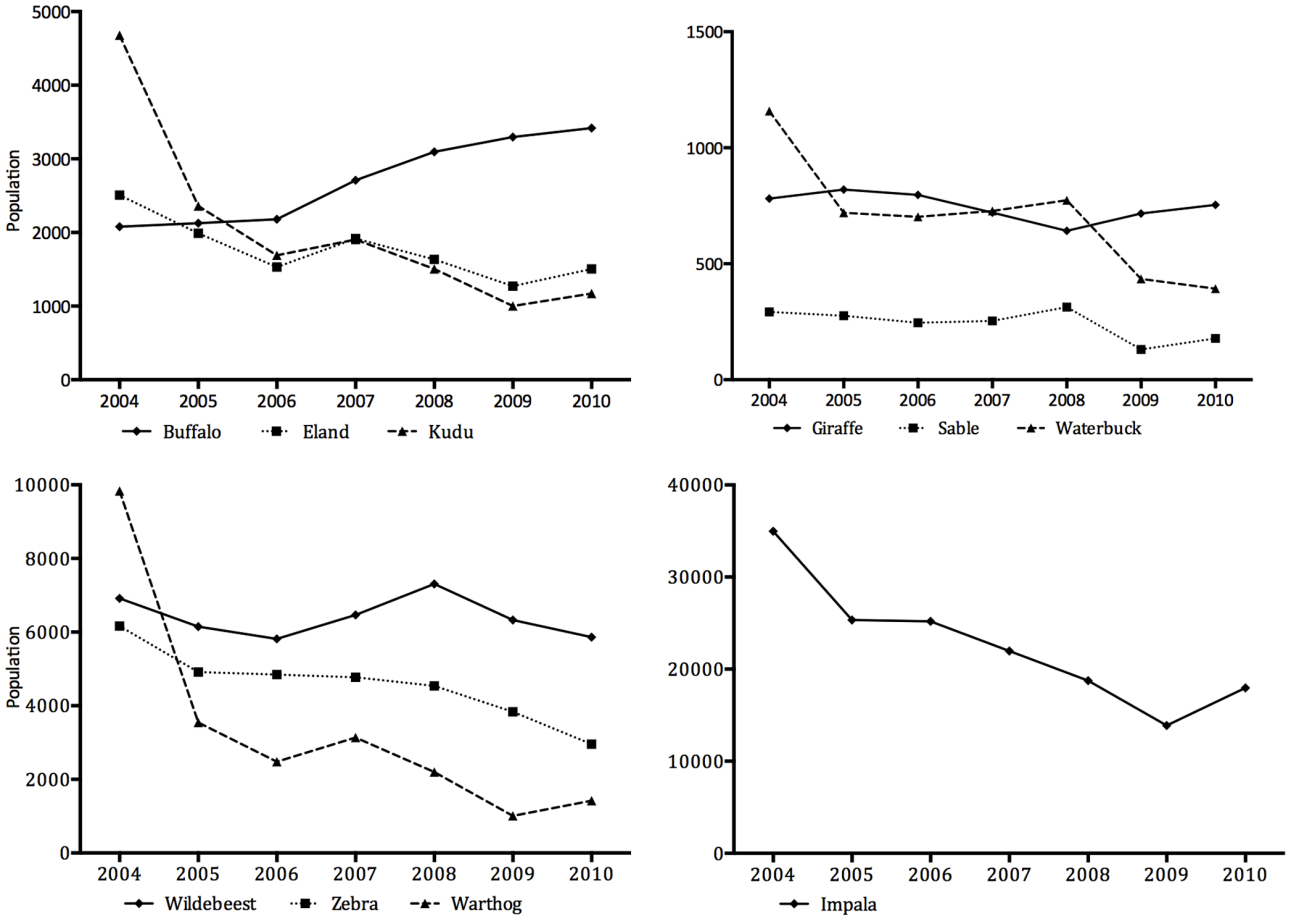
Population trends of ten key herbivore species in Savé Valley Conservancy from 2004 to 2010.

**Figure 2:**
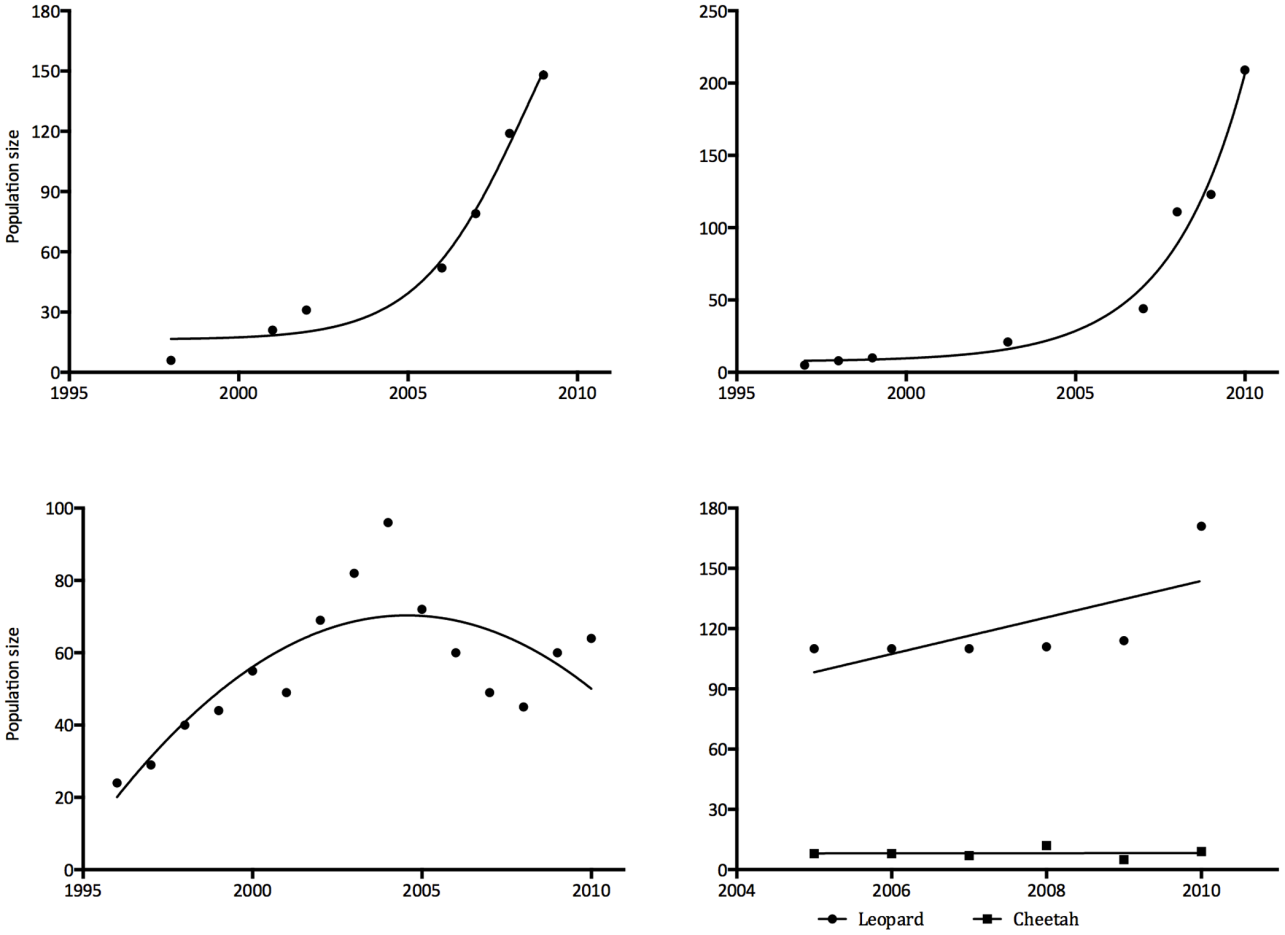
Population trends of a) lions, b) spotted hyenas, c) African wild dogs, and d) leopards (diamonds) and cheetahs (squares) in the Savé Valley Conservancy from 2000 to 2010.

Although hunted by humans and predators, buffalo increased during 2004–2010 (Fig. 1). By comparison giraffe (*Giraffa camelopardalis*), sable, and wildebeest (*Connochaetes taurinus*) remained relatively stable, and eland (*Taurotragus oryx*), kudu (*Tragelaphus strepsiceros*), waterbuck (*Kobus ellipsiprymnus*), zebra (*Equus burchelli*), impala (*Aepyceros melampus*) and warthog (*Phacochoerus aethiopicus*) numbers declined. Although most carnivores were present in SVC from 1996 the numbers of lions and spotted hyenas only started to increase markedly in 2006, whereas wild dogs increased to high numbers by 2003 and have declined slightly since then. Cheetah and leopard numbers appeared to be stable (Fig. 2).

### Large Predator Utilization

From 2005 to 2009 seventeen male lions were hunted (mean = 3.4 lions year^−1^), yielding gross income of US$912,900 (US$182,580 year^−1^). During the same time 144 leopards were hunted (mean = 28.8 leopards year^−1^), generating a gross income of US$2,135,232 (US$427,046 year^−1^).

### Financial Calculations

**Figure 3:**
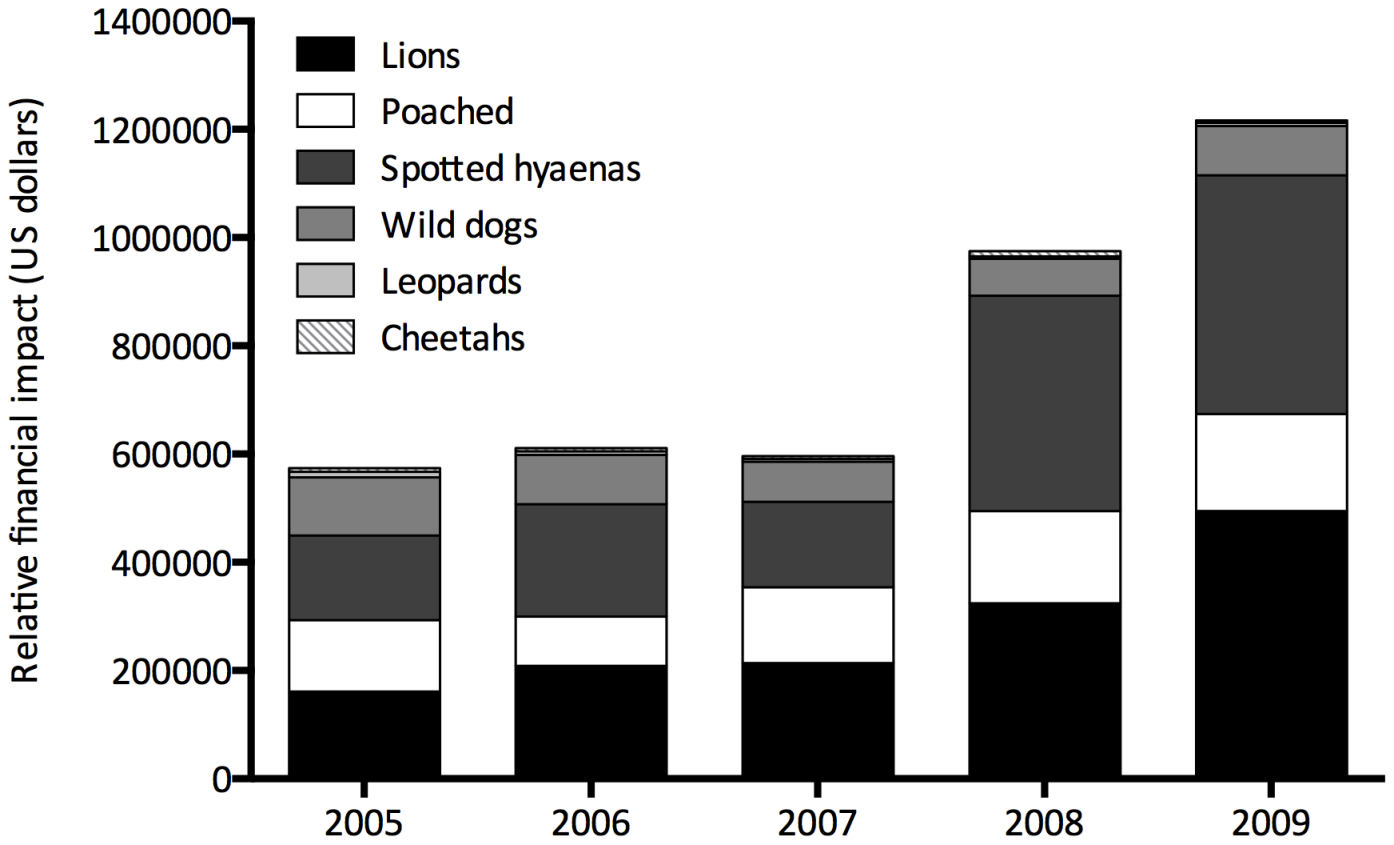
Stacked bar graph depicting the proportional economic losses in US dollars lost in the SVC to the five main predators and poaching from 2005 to 2009.

**Figure 4:**
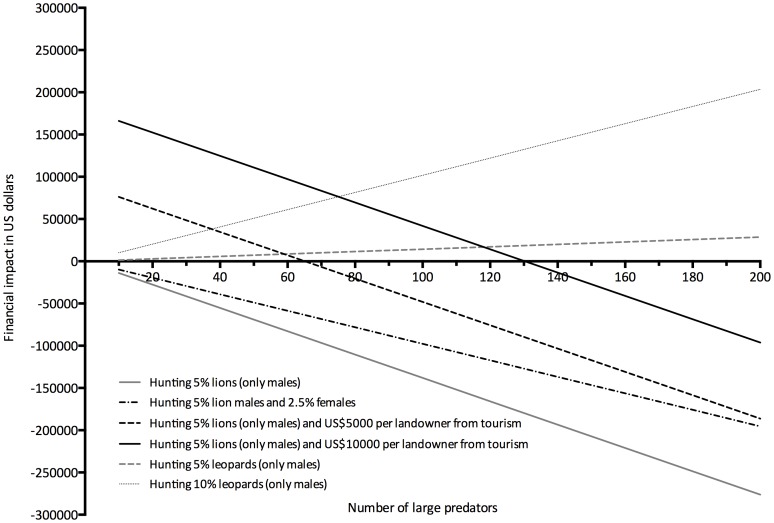
Projected net benefits of trophy hunting and photo-tourism based on 2009 trophy and day hunting fees for landowners in the Savé Valley Conservancy using various hunting off-take percentages. The numbers of individuals is displayed on the X-axis.

According to our model, lion predation, poaching and then spotted hyena predation imposed the greatest financial impacts, whereas the costs of leopards, wild dogs and cheetahs were much lower (Fig. 3). Furthermore, at all lion abundance levels the net cost of predation by lions exceeded the gross income obtainable from hunting them (Fig. 4). It should be noted though that gross income from trophy-hunting the selected herbivores, lions and leopards exceeded the financial losses to poaching and predation by an average of 74% (Table 1).

**Table 1:** The relative gross financial benefits (in US dollars) of trophy hunting ten key herbivores species, leopards and lions, relative to the costs incurred by poaching and predation.

	**2005**	**2006**	**2007**	**2008**	**2009**
**Hunting income:**					
Selected herbivores	1,689,670	2,104,260	2,11,8750	2,470,990	2,423,870
Lions	161,100	161,100	161,100	107,400	322,200
Leopards	400,356	504,152	504,152	370,700	355,872
**Total**	**2,251,126**	**2,769,512**	**2,784,002**	**2,949,090**	**3,101,942**
**Income lost to poaching (%)**	132,065 (5.9)	90,506 (3.3)	139,969 (5.0)	171,091 (5.8)	179,279 (5.8)
**Income lost to predators (%)**	437,670 (19.4)	496,561 (17.9)	457,874 (16.4)	693,357 (23.5)	906,450 (29.2)

Even relatively low earnings per landowner from photo-tourism (e.g. US$5,000 per year^−1^), combined with a 5% male lion harvest, resulted in a positive gross financial benefit for a population of up to 70 individuals. To balance out the predation effect of the current estimate of 160 lions, photo-tourism benefits per landowner would need to equal about US$ 13,000 year^−1^ (Fig. 4), or US$260,000 for the whole conservancy. However, there was a gross financial benefit at all levels of abundance for leopards (Fig. 4).

### Sensitivity Analysis

Manipulating the model in terms of the numbers of buffaloes eaten by more or less than 40% did not significantly influence the results of the model (Table 2). Similarly manipulating the percentages of predation on all other prey species, each one in turn by up to 40%, did not significantly influence the overall result.

**Table 2:** The relative financial costs (in US dollars) of lions when changing the relative predation rates on buffalo by as much as 40% (sensitivity analysis).

	2005	2006	2007	2008	2009
Cost of lion predation	161,084	208,930	213,989	323,950	494,776
10% less buffalo	162,112	207,029	215,119	325,661	497,074
20% less buffalo	165,348	206,047	201,297	387,654	496,908
30% less buffalo	159,498	205,550	200,398	397,603	497,603
40% less buffalo	159,342	205,488	195,467	398,540	485,376
10% more buffalo	161,158	208,937	214,009	323,999	494,789
20% more buffalo	163,009	208,978	215,101	324,089	495,901
30% more buffalo	164,078	209,012	216,172	325,200	497,999
40% more buffalo	166,158	209,089	218,354	326,357	498,156

## Discussion

Large predator numbers, especially lions, were the most important parameter in the model, and relative predation proportions were not a highly sensitive parameter in the model. Thus large predators influence ungulate populations in profound ways that managers need to monitor, and interpret in the context of predation and anthropogenic mortality and variation in rainfall [15], [16]. Positive growth of buffalos only, a high-value species, can probably be ascribed to good rainfall in preceding years, low predation rates by lions, and possibly lower trophy-hunting (conservative quotas were set for buffalo) and poaching mortality (buffalo are probably less vulnerable to snare poaching for meat) impacts. Sable antelopes, another high value species, seemed to be stable in number, and are not a favoured prey species for lions [17]. However, the more common ungulates, all of which are preferred by lions, trophy-hunters and poachers, were all in decline. Consequently, care is required by management when setting hunting quotas, implementing anti-poaching measures to avert population declines. Given that financial objectives are paramount in SVC consideration should be given to regulating large carnivore numbers. The sensitivity analyses demonstrated that the parameters in the model that affected income most significantly were the number of lions and the losses of ungulates to poaching. Thus managers in SVC need to be aware of the likely impact of growing lion numbers on ungulate populations and income from trophy hunting.

Using the models published by Hayward et al. [18] we estimated that the lion population in SVC is currently at about 60% of the expected ecological equilibrium. At these numbers the net financial impact of lion predation is no longer offset by the total income of trophy-hunting lions. This does not, however, account for the direct considerable potential for earnings associated with lions from other photo-tourism related ventures, nor the indirect marketing value associated with advertising hunting safaris in an area with “Big Five” species [19].

In contrast to lions, leopards resulted in a net financial benefit to SVC irrespective of their abundance levels. Lions thus impose a substantial financial cost to a wildlife production system (about 8% loss relative to gross income), which needs to be evaluated against the objectives of the area. In our model we assumed that 5% of the lion population would be hunted as trophies. Such levels of harvest may be excessive for populations that have reached carrying capacity, for which quotas of about 3% are recommended [37]. However, they may help limit further growth in the lion population. In wildlife areas where lion population estimates are not available, the impact of lion hunting on lion populations can be manipulated through imposing age limits on lion trophies [20]. However, published models on the impacts of harvest of lions of different ages [20] were based on stable populations at ecological capacity, and may not apply to a population that is growing exponentially. In the context of rapidly growing lion numbers, the harvest of either more or younger male lions, and possibly hunting some lionesses, may be needed to achieve effective population regulation [20], [21].

Wildlife-based land uses are typically viable by virtue of the fact that multiple use values can be derived from wildlife, including trophy hunting, meat, skins and photo-tourism [19]. However, in SVC, photo-tourism is currently not viable due to on going political instability, which greatly undermines the viability of wildlife-based land uses. Under such a situation, income (which is derived purely from trophy hunting) is likely to be sensitive to the density of large carnivores. Consequently, the numbers of dominant large predators, such as lions, may need to be controlled to ensure the on-going viability of wildlife-based land uses [4], [20], [21]. However, in situations whereby ecotourism is viable (such as will presumably arise in SVC when political stability returns to Zimbabwe), lions would likely become more of a financial asset and the optimal population level needed to achieve maximal earnings would be higher.

The situation in SVC is, however, not unique and thus our results apply to many other areas, such as large parts of Namibia [22] and other conservancies in Zimbabwe, South Africa and Zambia. For a combination of reasons, including for example lack of scenic splendour, lack of high densities of wildlife, relatively inaccessibility, lack of suitable facilities, and political instability, viable photo-tourism is limited to a minority of wildlife areas, meaning that most wildlife lands are reliant on trophy hunting for income. Furthermore, in many such areas, poaching for bushmeat is rampant. Thus, that the question of optimal densities for large predators, such as lions is widely relevant. Certainly in more developed continents such as Europe [23] and North America [21] the relative abundance of large carnivores is hotly debated, and very many large carnivores are killed each year in an attempt to achieve the precarious balance between productionist and conservation objectives.

Under a scenario of a managed lion population other large carnivores may temporally benefit. Both cheetahs [24], [25] and wild dogs [26], [27] have been recorded to be supressed by high lion and spotted hyena relative densities. These effects have implications for attempts to conserve smaller predators both generally, and through reintroduction programs [28]. Wild dogs in SVC comprise a significant component (c. 17%) of a small and declining national population, and the recent population declines in the conservancy are of concern. In such a scenario, management intervention to control lion populations as a means of enhancing the prospects of effective conservation of rarer and competitively inferior species may be justifiable [21], [29].

## Materials and Methods

### Study Area

The 3,440 km^2^ Savé Valley Conservancy (SVC) is located in southeast Zimbabwe in an area of semi-arid savanna typified by low and variable rainfall [30]. SVC was constitutionally inorgreated in June 1991, and following a severe drought in 1991/1992 wildlife ranching became the primary landuse. At the time this was the largest private wildlife conservancy in the world, being comprised of 18 former cattle ranches under a largely unified management structure. After formation of the conservancy, significant investments were made in tourism infrastructure, external perimeter fencing and the reintroduction of∼4,000 individuals of 14 herbivore species [31]. A land reform program was initiated in Zimbabwe in February 2000 whereby farms where taken from largely white farmers and given to black people. The ensuing political instability resulted in a collapse of the photo-tourism industry, resulting in trophy hunting becoming the only economically viable land use option. Today SVC relies on almost exclusively on the sale of trophy hunting safaris to high-paying foreign tourists for income. High value species such as lions, leopards, elephants (*Loxodonta africana*), African buffalo (*Syncerus caffer*) and sable (*Hippotragus niger*) contribute the bulk of the revenues generated from hunting, although ungulates are also extensively hunted [11]. Due to the on going political instability, SVC experiences severe bush-meat poaching which threatens the viability of trophy hunting enterprises [11].

### Population Estimates

Prior to the establishment of SVC, large predators were virtually eradicated from the area. Lions and wild dogs were eradicated, cheetahs (*Acinonyx jubatus*) and spotted hyenas (*Crocuta crocuta*), with only leopards persisting in reasonable numbers [30], [32]. Since the formation of the conservancy, the species already resident increased in number, and wild dogs and lions recolonized the area naturally. Both species were present on SVC by 1996, and although 13 lions where introduced by 2003, wild dog numbers initially recovered much more quickly than the lions did [31]. Wild dog numbers increased rapidly, but lions and spotted hyenas took longer to recover. From 2007 large predator numbers were estimated annually by track index surveys [33]. Prior to this lion and spotted hyena populations were estimated sporadically with call-up surveys [33]. Wild dog numbers are known from direct monitoring. Herbivore numbers are estimated annually using total-count aerial surveys [34], adjusted for undercounting [35].

### Model Development

We developed a model to assess the net financial impacts of predators in SVC. The modelling process followed two key steps. First we built a biological model to estimate the numbers of prey species that would have been killed by the complement of predators estimated [4], [36] and then used those results to estimate financial outcomes. In the financial model we assessed the impact of all forms of mortality on ranch finances. We set maximum lion harvesting rates at 5% as rates of 3% are thought to be sustainable [37], although here slightly higher harvest rates would have been acceptable to limit population growth. Key inputs of the respective models included:

#### Prey preferences of large predators

To determine the relative proportion of the various ungulate species in the large predator diet we used scat analysis data from SVC for leopards, wild dogs and spotted hyenas [38]. Low numbers of lion and cheetah scats necessitated using data published from Kruger National Park [39], [40], [41]. The habitats and ungulate assemblages in Kruger and SVC are similar [30], [31], [32], [39], [40], [41] with the densities of lions in northern Kruger being similar to that in SVC [42].

#### Predation rates

To estimate annual predation rates we determined the daily minimum food requirements for each predator species from literature [32], [43], [44], [45], [46]. We then calculated the number of adult female equivalents, based on body mass [47], of each herbivore needed to meet those needs, in proportion to the prey preference ratios [above]. The biomass available per kill was based on an estimate of the relative proportion of meat available from small- [<100 kg = 75%], medium- [100–300 kg = 66%] and large-sized [>300 kg = 60%] ungulates. Although increasing populations have a high percentage of juveniles, subadult lions eat more than adults [39]. Thus in our model adults were assumed to make up 50% of each large predator population, and sub-adults and cubs were assumed to consume 50% of the adult female minimum daily requirements [32], [43], [44], [45], [46]. Due to differences in the diets of males and females we calculated sex specific predation rates for lions [39], [40]. Although male lions scavenged more than females did in Kruger National Park [39], most lions scavenged from the kills of other lions. Thus we did not account for scavenging by lions in the biological model.

The total numbers of ten most abundant small [50–10 k kg] and medium to large [>100 kg] herbivore prey species [excluding megaherbivores] killed per large predator species per year were estimated by combining the relative proportions of each herbivore in the diet, the biomass requirements of each large predator, and the numbers each prey species that an individual would likely have killed, multiplied by the annual population estimate of each large predator species (see Table 3). This approach has been successfully followed with other similar studies [4], [5]. We halved this for spotted hyenas as in Kruger National Park spotted hyenas scavenged 50% of their food [48], and we could not find any reason to believe that this should not translate to the similar conditions in SVC.

**Table 3:** Percentage of herbivores in the diet of the large predators used in the development of the model, with the estimated number of each key ungulate species caught per individual predator per year.

Prey size/species	Percentage of each prey species (number of prey species caught year^−1^)					
	Male lions	Female lions	Leopards	Cheetahs	Wild dogs	Spotted hyenas
**Large (>350 kg)**						
Buffalo	45.0 (4.50)	1.2 (1.20)	0.0 (0.00)	0.1 (0.02)	−	2.6 (0.05)
Eland	4.0 (1.00)	4.5 (0.22)	0.0 (0.05)	−	−	1.3 (0.03)
Giraffe	19.3 (1.10)	4.3 (0.12)	−	−	−	−
**Medium (100–350 kg)**						
Kudu	1.4 (0.40)	9.3 (1.23)	7.5 (0.50)	3.7 (0.23)	12.5 (1.16)	19.2 (1.01)
Sable	1.1 (0.20)	2.0 (0.23)	0.1 (0.35)	−	0.1 (0.11)	6.4 (0.20)
Waterbuck	1.4 (0.30)	3.1 (0.36)	1.5 (0.26)	1.2 (0.07)	4.2 (0.34)	2.6 (0.12)
Wildebeest	2.1 (0.50)	10.9 (1.17)	6.0 (0.32)	2.7 (0.14)	1.4 (0.11)	6.4 (0.28)
Zebra	6.4 (0.90)	8.4 (0.58)	−	−	−	−
**Small (<100 kg)**						
Impala	13.6 (12.10)	40.2 (17.94)	60.3 (14.99)	67 (14.50)	74 (16.46)	46 (8.25)
Warthog	5.7 (3.70)	11.3 (3.65)	7.5 (0.24)	4.2 (0.65)	2.8 (0.63)	−
**Other**	0.0	1.2	17.2	20.6	5.5	15.3

#### Determining ungulate population trends

We estimated mortality by summing all quantifiable causes of ungulate mortality. The number of animals trophy hunted was determined from hunting returns. The number of animals found poached was recorded during a recent study, and adjusted upwards by 50% for under-recording of illegal off-takes [11].

#### Economic calculations

Financial returns from trophy hunting were calculated as the trophy fee and the meat value of each animal hunted. The financial losses attributable to predation and poaching were calculated by summing the value lost in terms of meat, foregone trophy fees, and for buffalo and sable for foregone hunt-package fees. All trophy values, daily rates and minimum hunt durations were taken from the main hunting operator in the area [49]. The value of meat was assumed to be USD 1.50 kg^−1^. Since not all herbivores lost to predation or poaching would have been trophies, only the percentage of these taken by hunters [averaged from 2005–2009] was assumed to result in the loss of otherwise saleable trophies.

#### Sensitivity analysis

Modelling is a compromise between the need for maximum realism and minimum complication in construction and design [50]. As it is impossible to achieve absolute realism, especially with limited data, a number of simplifications and assumptions have to be incorporated into the process. A sensitivity analysis was performed to assess which input parameters had the greatest effect on the model outputs. This was done by either increasing or decreasing the parameters by 10, 20, 30 and 40% [51]. We were particularly interested in investigating the effect of changing predation rates on buffalo, as they are a high value species relatively favoured by lions for which we did not have SVC-specific data.

### Conclusions

Lion hunting is an important income generator for Zimbabwe, and generally in African hunting concessions [2], [52]. Thus to facilitate wildlife-based land uses that compete with alternative options (e.g. livestock ranching or agriculture) it is important that landholders and communities are able to utilize wildlife to offset the costs of wildlife. This may include well managed sustainable trophy hunting [19], which in many areas is the only viable means of generating income from wildlife [52]. In private or communally owned wildlife areas, management of larger predators may be necessary to maximize other returns [21]. This may in turn create favourable opportunities that ensure the persistence of endangered large predators, and may even be justifiable on these grounds alone in some small state protected areas. Our findings may thus be limited to commercial game ranches, and should not necessarily applied to areas where biodiversity conservation is the primary objective, even if hunting is allowed there.
